# Effect of Vitamin D on Experimental Autoimmune Neuroinflammation Is Dependent on Haplotypes Comprising Naturally Occurring Allelic Variants of CIITA (*Mhc2ta*)

**DOI:** 10.3389/fneur.2020.600401

**Published:** 2020-11-13

**Authors:** Sonja Hochmeister, Shahin Aeinehband, Charles Dorris, Rasmus Berglund, Michaela T. Haindl, Vid Velikic, Sven A. Gustafsson, Tomas Olsson, Fredrik Piehl, Maja Jagodic, Manuel Zeitelhofer, Milena Z. Adzemovic

**Affiliations:** ^1^Department of General Neurology, Medical University of Graz, Graz, Austria; ^2^Department of Clinical Neuroscience, Center for Molecular Medicine, Karolinska Institutet, Stockholm, Sweden; ^3^School of Biosciences and Medicine, University of Surrey, Guildford, United Kingdom; ^4^Clinical Division of Social Psychiatry, Department of Psychiatry and Psychotherapy, Medical University of Vienna, Vienna, Austria; ^5^Department of Molecular Medicine and Surgery, Clinical Chemistry and Blood Coagulation Research, Karolinska Institutet, Stockholm, Sweden; ^6^Vascular Biology Unit, Department of Medical Biochemistry and Biophysics, Karolinska Institutet, Stockholm, Sweden

**Keywords:** CIITA (Mhc2ta), vitamin D, experimental autoimmune encephalomyelitis (EAE), multiple sclerosis (MS), allelic variant

## Abstract

An increasing body of evidence associates low vitamin D levels with increased risk of multiple sclerosis (MS), suggesting the possibility of a gene-environment interaction for this environmental factor in MS pathogenesis. Moreover, it has been shown that vitamin D downregulates major histocompatibility complex (MHC) class II expression in experimental autoimmune encephalomyelitis (EAE), an animal model of MS. We here report about the impact of a dietary vitamin D supplementation on EAE in the rat strains having functionally relevant allelic variations in the *CIITA* (*Mhc2ta*) gene, a master regulator of MHC class II expression. Full length myelin oligodendrocyte glycoprotein (MOG)-EAE was induced in DA.PVG^av1^-*Vra4* congenic rats harboring the *Vra4* locus from PVG strain in the EAE- susceptible DA background, and compared to the parental strains. The congenic rats fed with either vitamin D supplemented, deprived or regular diet developed an intermediate clinical EAE phenotype, in contrast to DA and PVG strains. Immunopathological studies revealed vitamin D dose-dependent effect on demyelination and inflammatory infiltration of the central nervous system (CNS), expression of MHC class II and CIITA, as well as downregulation of a range of pro-inflammatory genes. Taken together, our findings demonstrate an impact of vitamin D on the target tissue pathology and peripheral immune response during EAE in DA.PVG^av1^-*Vra4* congenic strain. Thereby, our data provide evidence of a modulatory effect of vitamin D in context of genetic variances in the *Vra4* locus/*Mhc2ta* gene in MS-like neuroinflammation, with potential relevance for the human demyelinating disease.

## Introduction

Numerous studies implicate gene-environment interactions in the complex pathogenesis of multiple sclerosis (MS) (1). Allelic variations in the human leucocyte antigen (HLA) system, a group of genes on chromosome 6p21 that serves as the major histocompatibility complex (MHC), have been shown to enhance the probability for developing MS (2, 3). Polymorphisms in this locus have also been implicated in development of other autoimmune diseases such as Type I diabetes (4, 5), systemic lupus erythematosus (6), rheumatoid arthritis (RA) (7, 8) and narcolepsy (9). *CIITA* (*Mhc2ta*) gene encodes class II transactivator, shown to be essential for MHC class II transcription and expression (10). An association between a genomic region containing *Mhc2ta* and other MS candidate genes *Clec16a, Dexi*, and *Socs1* (11) and an increased risk for MS, RA, and myocardial infarction indicates correlation between quantitative aspect of MHC class II and susceptibility to MS (12).

Similarly, distinct susceptibility of rodent strains to experimental autoimmune encephalomyelitis (EAE), an animal model of MS, has been linked to different variants of MHC class II region (referred to as RT1 in the rat) coding for MHC class II molecules (13). Differences in genetically regulated MHC class II phenotype resulted in classification of inbred rat strains into high and low responders to EAE (14). Further, *Vra4* locus was recognized as a regulator of MHC class II upon mechanical nerve injury in the F2 intercross between high (Dark Agouti; DA) and low (Piebald-Viral-Glaxo; PVG) responder strains (15). The locus was then fine-mapped in an advanced intercross line, which led to identification of the candidate gene *Mhc2ta* (12). Further study from our lab demonstrated less severe EAE in the congenic DA.PVG^av1^-*Vra4* rats compared to the susceptible DA strain, which was primarily attributed to the *Vra4* allele-dependent quantitative differences in MHC class II expression (15).

Vitamin D response elements (VDREs) have been detected in the promoter region of the MS-associated allele HLA-DRB1^*^15:01 (16) indicating an interplay between genetic and environmental factors in pathogenesis of the demyelinating disease (17). MS patients commonly have a history of reduced serum levels of vitamin D (18), which is linked to certain life-style and environmental factors (19). However, allelic variants of vitamin D receptor (VDR) and VDREs in different human populations may determine the threshold for vitamin D deficiency and thus risk for MS (20).

Promoter region of the 1,25-(OH)_2_D-24-hydroxylase gene in rat contains the most powerful vitamin D-responsive element reported to date (21), which underlines relevance of rodent models for studying vitamin D-related modulation of autoimmune diseases. We have previously reported an age-dependent efficacy of vitamin D in ameliorating MS-like neuroinflammation in DA rats and identified juvenile/adolescent age as a time-window for the most efficient preventative treatment (22, 23). Here we demonstrate that the same dietary supplementation had a different effect on EAE in juvenile/adolescent DA.PVG^av1^-*Vra4* congenic rats sharing genetic background with DA, apart from the *Vra4* fragment that originates from PVG^av1^. Our data emphasize the relevance of *Vra4* haplotype/*Mhc2ta* variants for vitamin D-mediated modulation of EAE.

## Materials and Methods

### Ethical Statement

The experiments were performed in accordance with the guidelines from the Swedish National Board for Laboratory Animals and the European Community Council Directive (2010/63/EU) under ethical permits approved by the North Stockholm Animal Ethics Committee (Stockholms Norra djurförsöksetiska nämnd). Experimental animals were housed in the animal facility at Karolinska University Hospital (Stockholm, Sweden). They were tested according to a health-monitoring program at the National Veterinary Institute (Statens Veterinärmedicinska Anstalt, SVA) in Uppsala, Sweden.

### Experimental Animals and Diet Regimen

All rats were housed in a specific pathogen-free and climate-controlled environment in polystyrene cages containing aspen wood shavings with free access to rodent chow and water with regulated 12-h light/dark cycles free of UV radiation.

Congenic DA.PVG^av1^-*Vra4* rats were generated in our lab as previously described (15) by transferring the *Vra4* fragment from PVG onto DA background. The original *Vra4* male donors were selected from the G8 generation of a DAxPVG^av1^ advanced intercross line for transferring a small, well-defined fragment harboring the *Vra4* congenic region (RNO10: 0-D10Mit14). Repeated backcrossing to the respective recipient strain was performed for an additional nine generations to create congenic rats with theoretically <0.1% of the donor genome outside the *Vra4* locus.

Diet groups based on different contents of vitamin D_3_ (cholecalciferol; referred to as vitamin D) were described in detail in our previous study (23). In this study, newly weaned (3 weeks-old) congenic DA.PVG^av1^-*Vra4* (*n* = 11-12), DA (*n* = 11) and PVG (*n* = 12) male and female pups were split into three groups and subjected to either: (i) supplemented diet containing 10 International Units/g (IU/g) of vitamin D, which is five-fold increased amount of the vitamin comparing to the regular rat diet; (ii) regular rat diet containing 2 IU/g of vitamin D; or (iii) vitamin D deprived diet (0 IU/g vitamin D). Animals were subjected to the diets five weeks prior to immunization and continuously until the end of the EAE experiment. The supplemented and deprived diet formulations were obtained from TestDiet Limited (London, UK).

### Immunization and EAE Phenotyping

Five weeks after the diet regime was initiated, animals were immunized to induce EAE. Myelin oligodendrocyte glycoprotein (MOG, amino acids 1–125 from the N terminus) used for inducing EAE was expressed in Escherichia coli and purified to homogeneity by chelate chromatography (24). During immunization procedure, rats were anesthetized using isoflurane (2-chloro-2-(difluoromethoxy)-1,1,1-trifluoro-ethane, Forene, Abbott Laboratories, Chicago, IL, USA). 200 μL inoculum injected subcutaneously in the tail base contained 15 μg MOG in phosphate buffered saline (PBS), emulsified 1:1 with incomplete Freund's adjuvant (Sigma-Aldrich, St. Louis, MO, USA). All animals were monitored daily for clinical signs of EAE, starting on the day 7 post immunization (p.i), until the end point. EAE phenotyping was performed as follows: 0 = no detectable clinical signs; 1 = tail weakness or paralysis; 2 = hind limb hemiparesis or paraparesis; 3 = hind limb paralysis and 4 = tetraplegy or moribund. EAE course was evaluated through the disease parameters: disease onset (the first day of clinical disease manifestation), maximum EAE score (the highest clinical score during entire disease course), cumulative EAE score (the sum of daily clinical scores), and duration of EAE (amount of days with clinical manifestation).

### Analysis of the Vitamin D Serum Levels

Effect of the diet regimens on serum levels of 25-hydroxy vitamin D (25(OH)D) (*n* = 6 rats per diet group) were estimated on day 7 p.i using a chemiluminescent immunoassay from Diasorin (Diasorin AB, Sundbyberg, Sweden) and a LIAISON® instrument provided by Diasorin ABwith equimolar measurement of both 25-OH vitamin D_2_ and D_3_. The serum was purified from blood according to standard procedures, subsequently frozen in liquid nitrogen and stored at −80°C until used for the analysis.

### FACS Analysis

The analyses (*n* = 12 rats per diet group) were performed on single cell suspensions (SCS) obtained from the local draining inguinal lymph nodes harvested 7 days p.i. (d7 LN). A triple staining procedure was performed using: CD3-FITC, CD4-APC, and CD45RA-PE (BD Biosciences, San Jose, CA, USA, 1:100, for 30 min at 4°C). Target- and control staining were visualized using a FACS Calibur (BD, Franklin Lakes, USA) with CellQuest software (version 3.2.1f1, BD) and analysed using Kaluza software (Beckman Coulter, USA).

### Quantitative Real-Time PCR

The analyses (*n* = 8-12 rats per diet group) were performed on whole d7 LN harvested on day 7 p.i. Total RNA was extracted from the SCS using the RNeasy kit (Qiagen, Hilden, Germany) and the QIAcube (Qiagen) including on column DNA-digestion for fully automated sample preparation. RNA concentration and purity was determined through measurement of A260/A280 ratio with a NanoDrop ND-1000 Spectrophotometer (NanoDrop Technologies, Wilmington, DE, USA). Confirmation of RNA quality was assessed using the Agilent 2100 Bioanalyzer (Agilent Technologies, Santa Clara, CA, USA). cDNA was prepared using the iScript kit (Bio-Rad, Hercules, CA, USA). Quantitative real-time PCR (qPCR) was performed using a CFX384 touch real time PCR detection system (Bio-Rad) with a two-step PCR protocol (95°C for 10 min followed by 40 cycles at 95°C for 10 s and 60°C for 30 s and 45 cycles of melt curve analysis), using SYBR Green (Bio-Rad) as the fluorophore. Relative expression levels, corrected for amplification efficiency, were analyzed using the CFX manager software (Bio-Rad). Relative expression was calculated as the ratio between the target and *Rpl19*. Serial 10-fold dilutions from a pool of undiluted samples within the study were used as standard. The following primers were used for SYBR Green: *Rpl19*: GGA CCC CAA TGA AAC CAA CG and AAG GTG TTC TTC CGG CAT CG; *Il2*: CGA TAA TT A CAA GAA TCT GAA ACT CC and TTT CCA GCG TCT TCC AAG TG; *Il4*: CTT ACG GCA ACA AGG AAC ACC and CTT TCA GTG TTG TGA GCG TGG; *Icam1*: GCA GAT GGT GTC CCG CTG CC and CCA CGA TCA CGA AGC CCG CA; *Ifng*: AAA GAC AAC CAG GCC ATC AGC and TGG CGA TGC TCA TGA ATG C; *Mhc2ta*: CAT ACT CTG TGT GCC ACC ATG G, and AGT TCG ATC TCT TCC TCC CCA; *Cd4*: AAG GAC TGG CCA GAG ACT CAG AT and ACG ACT ATA CAG CTC AAG TGA ACC; *Tgfb*: CAA TTC CTG GCG TTA CCT TGG and AAA GCC CTG TAT TCC GTC TCC; *Stat6:* TGT CAC CGA AGA GAA GTG CG and TGA ACG ATG ACC ACC AAG GG; Gata3: CAC GAT CCA GCA CAG AAG GC and GGT CTC CGT TAG CGT TCC TC; *Rorc*. TTT GAA GGC AAA TAC GGT GGT G and TGT CGA TGA GTC TTG CAG AGA TG; *Il17a*: GAC CTA GAA GGA CCG TTG TGC and TTA GGG TGA AGC ATT CCC TGC; *MhcII:* AGA AAC AGC AAG CCA GTC and GGA TGA AGG TGA GGT AAG C.

### Histopathology and Immunotargeting

On day 33 p.i, animals from each diet regimen (*n* = 4–6) were euthanized using CO2 and perfused via the left heart ventricle with PBS followed by 4% paraformaldehyde. Paraformaldehyde-fixed 3–5 mm thick paraffin embedded sections of the rat central nervous system (CNS) were dewaxed in xylol, rehydrated and then stained with hematoxylin and eosin and Luxol Fast Blue (LFB) to assess tissue inflammation and demyelination, respectively. The inflammatory index (I.I.) and demyelination score (DM) were determined from the number and size of demyelinated lesions in each animal on an average of eighteen complete spinal cord cross-sections as previously described (25).

For immunohistochemistry (IHC) and immunofluorescence (IF), adjacent spinal cord sections were treated as previously described (26). After antigen retrieval, sections were incubated with 10% fetal calf serum (FCS) in 0.1 mol/L PBS prior to incubation with primary antibody (at 4°C, overnight) against the following targets, respectively: lysosomal membrane of activated macrophages and microglia cells (ED1, mouse anti-rat monoclonal antibody, AbD Serotec, Germany; 1:1000), rat T lymphocytes (W3/13, mouse anti-rat, Abcam, 1:100), anti-amyloid precursor protein (APP, mouse anti-rat, Millipore Chemikon, 1:500). Co-staining was performed against CIITA (rabbit anti-rat, LSBio, 1:200) and MHC class II RT1B (mouse anti-rat, AbD Serotec, 1:200). Control sections were incubated in FCS in absence of the primary antibody. For IHC, sections were subsequently washed in PBS and then incubated with biotinylated secondary antibody in 10% FCS in PBS for 1 hour (h) at room temperature (RT; anti-mouse or -rabbit, Amersham Pharmacia Biotech, 1:200). Finally, avidin peroxidase (1:100, Sigma) was applied for 1 h at RT and subsequently visualized with 3,3′diaminobenzidine-tetrahydrochloride (DAB, Sigma). For IF, after washing in PBS, sections were incubated with fluorescently labelled secondary antibodies [alexa488 (1:1000) and alexa555 (1:1000)], including DAPI (1:2500). As for histopathology, evaluation of immunotargeting was performed on an average of eighteen whole spinal cord cross-sections using either Leica Polyvar 2 or confocal microscope Zeiss LSM 700.

### Statistical Analysis

Statistical analyses of the clinical EAE parameters were performed using non-parametric Kruskal–Wallis test followed by Dunn's multiple comparison test for all analysed pairs/groups. Statistical analyses of 25(OH)D serum levels, FACS, histopathology, IHC, and qPCR were performed using one-way ANOVA with Tukey correction for multiple testing for all analyzed pairs/groups. Sample size and statistical analysis methods are given in each figure for every experimental setup.

## Results

### Vitamin D Has a Different Effect on the Course of EAE in DA.PVGav1-*Vra4* Congenic Rats Comparing to the Parental Strains

**Figure 1:**
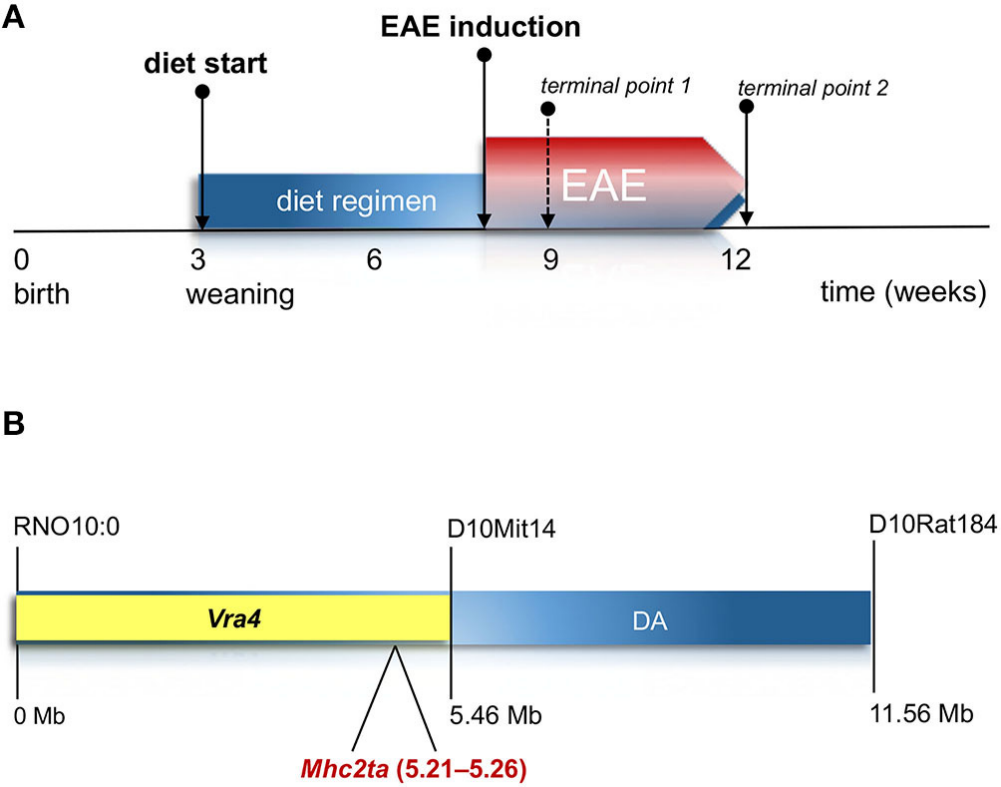
Experimental design and *Vra4* congenic region. **(A)** Experimental setup and diet regime based on different contents of vitamin D in rodent chow. 3-week-old newly-weaned female and male pups were assigned into three groups and subjected to either: (i) vitamin D deprived diet (0 IU/g); (ii) diet containing regular amount of vitamin D (2 IU/g), or (iii) vitamin D supplemented diet (10 IU/g). EAE was induced 5 weeks after initiation of the diet regimen, and lasted from 7 up to 40 days p.i., when the animals have been sacrificed for diverse analyses. **(B)** *Vra4* congenic region on rat chromosome 10. The DA.PVGav1-*Vra4* congenic strain harbors *Vra4* fragment from PVG^av1^ in the DA background. *Vra4* is defined as a 5.3-Mb genomic region with the borders at which the lod score drops by 1.5 relative to the maximum linkage support in the quantitative-trait locus (QTL). The congenic region contains the candidate gene *Mhc2ta* close to the maximum lod peak. The PVG fragment in our DA. PVG^av1^-*Vra4* congenic strain spans from the acrocentric region of chromosome 10 to the marker D10Mit14.

**Figure 2:**
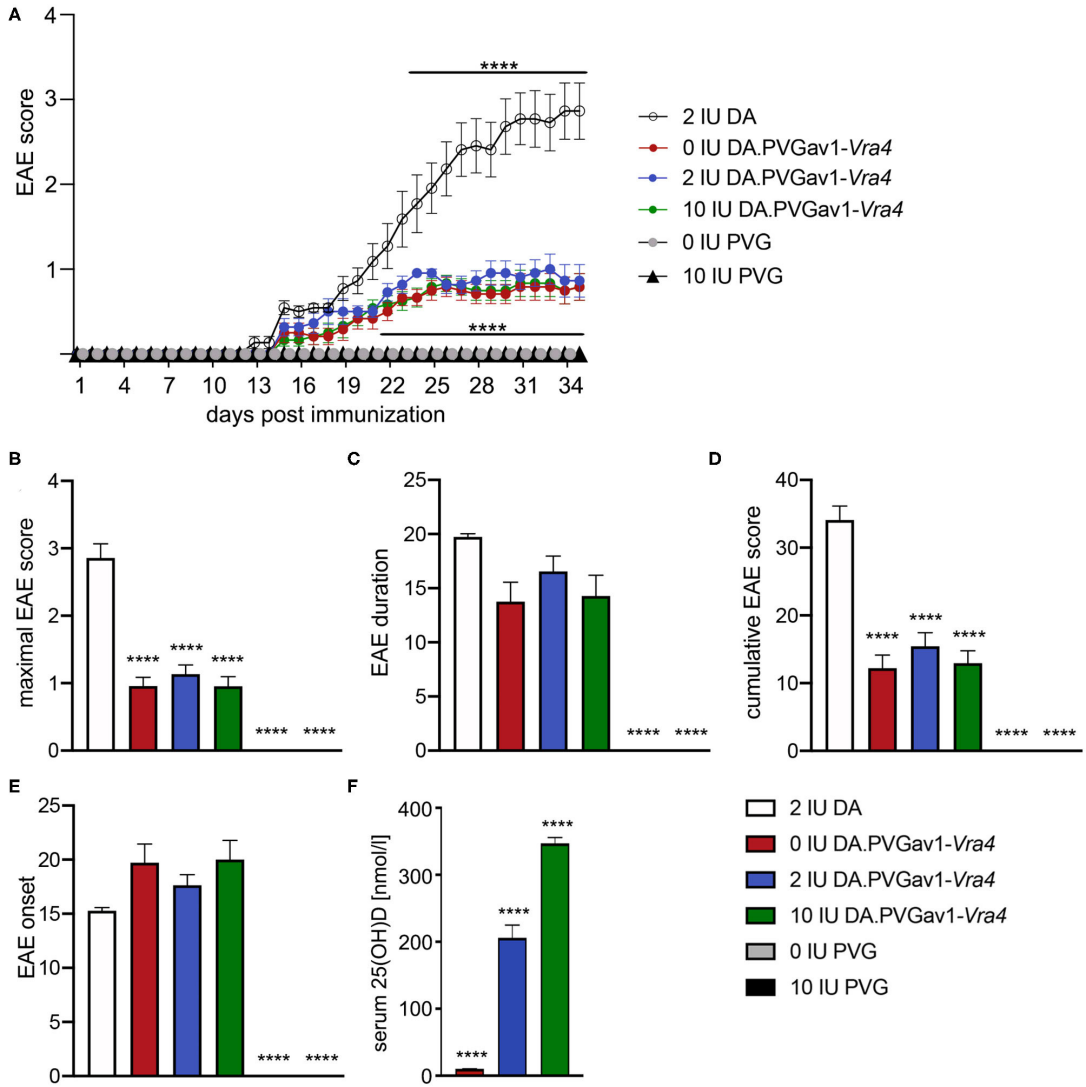
Impact of vitamin D on the course of EAE in DA.PVGav1-*Vra4* congenic and parental strains. **(A)** DA rats (*n* = 11) developed expectedly severe disease course, whereas PVG rats subjected either to vitamin D supplemented (*n* = 12) or deprived diet (*n* = 12) did not develop any signs of the disease. No additional modulation of essentially mild disease course in the congenic strain upon different concentrations of vitamin D (vitamin D supplemented (*n* = 12), vitamin D deprived (*n* = 12) and the regular diet group (*n* = 11)); both sexes included; data based on 2 pooled, independent experiments with comparable outcome. **(B–E)** Maximal EAE score, EAE duration, cumulative EAE score and EAE onset were comparable between the diet groups. **(F)** Serum levels of 25(OH)D were significantly different between the diet groups measured day 7 p.i. (*n* = 6 per diet group). Error bars represent SEM. Statistics were calculated using Kruskal–Wallis test with Dunn's correction for multiple testing (****p < 0.0001) for A and one-way ANOVA with Tukey correction for multiple testing (****p < 0.0001) for B-F. Statistical significances refer to comparison between DA.PVGav1-*Vra4* and DA or DA.PVGav1-*Vra4* and PVG, respectively.

In order to investigate the impact of vitamin D on MS-like neuroinflammation in the context of *Vra4* haplotype/*Mhc2ta* variants, DA.PVG^av1^-*Vra4* congenic rats were subjected to previously established diet regimen based on either: (i) regular, (ii) vitamin D supplemented, or (iii) vitamin D deprived rodent chow (23) (Figure 1A). An earlier study from our lab demonstrated that DA.PVG^av1^-*Vra4* rats harboring *Vra4* congenic region descending from the PVG strain (Figure 1B) develop significantly milder EAE than the susceptible recipient DA strain (15). Notably, we could observe that regardless of the vitamin D concentration in their diet, congenic rats could develop clinical disease accompanied by histopathological features typical for MOG-induced EAE, including involvement of MHC (13, 25, 27). However, clinical EAE was essentially mild and comparable in all three diet groups, illustrated by similar maximal and cumulative scores, as well as onset and duration of the disease (Figures 2A–E). Notably, concomitantly immunized age-matched DA rats subjected to the regular diet developed expectedly severe disease course (Figure 2A), while PVG strain remained unsusceptible to EAE regardless of the vitamin D concentration in the chow. Efficacy of the diet regimen in the congenic strain was verified by measuring serum levels of 25(OH)D, which were the highest in the supplemented group (Figure 2F).

### Vitamin D Modulates CNS Pathology in the Congenic Strain

**Figure 3:**
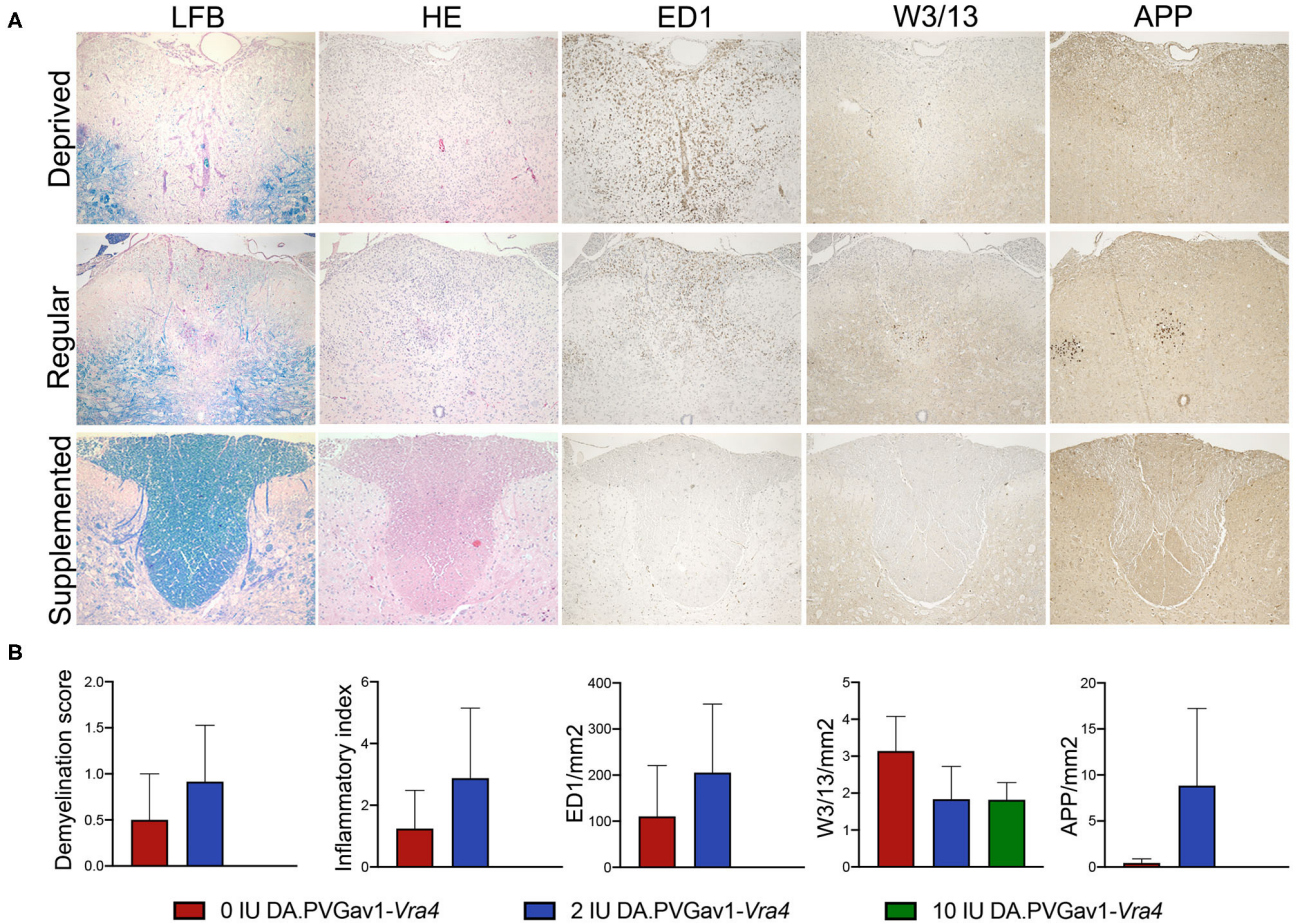
CNS inflammation and demyelination in the congenic rats upon different concentration of vitamin D in the diet. **(A)** Images of the spinal cord cross-sections of DA.PVGav1-*Vra4* rats subjected to different levels of vitamin D in their diet. Histopathological and IHC analyses were performed on an average of 18 paraffin embedded spinal cord cross-sections per animal harvested on day 33 p.i. (*n* = 4- 6 per diet group). The sections were stained with LFB, HE, against ED1, W3/13, and APP, respectively, in order to assess: **(B)** demyelination score (DM), inflammatory index (I.I.), recruitment of ED1+ microglia/macrophages, W3/13+ lymphocytes, and axonal damage, respectively. Statistics were calculated using one-way ANOVA with Tukey correction for multiple testing.

Histopathological analyses revealed that congenic rats subjected to vitamin D supplemented diet did not have any signs of neuroinflammation and demyelination left in the CNS 33 days p.i., in contrast to the groups fed with either regular or vitamin D deprived diet (Figure 3). Remaining inflammatory demyelination in some animals of the latter two diet groups was accompanied by ED1+ activated microglia/macrophages and axonal damage-associated APP aggregates, which were no longer present in the supplemented group. Interestingly, we were still able to detect T lymphocytes in all three diet groups on day 33 p.i., nevertheless, a tendency toward lower infiltration was detected in the group subjected to the vitamin D supplemented diet. These observations indicate association between vitamin D-mediated impact on immune cell recruitment and more effective recovery of the CNS parenchyma in the congenic strain.

### Vitamin D Downregulates Peripheral Pro-inflammatory Immune Response and Modulates Expression of MHC Class II and CIITA in the CNS of the Congenic Strain

**Figure 4:**
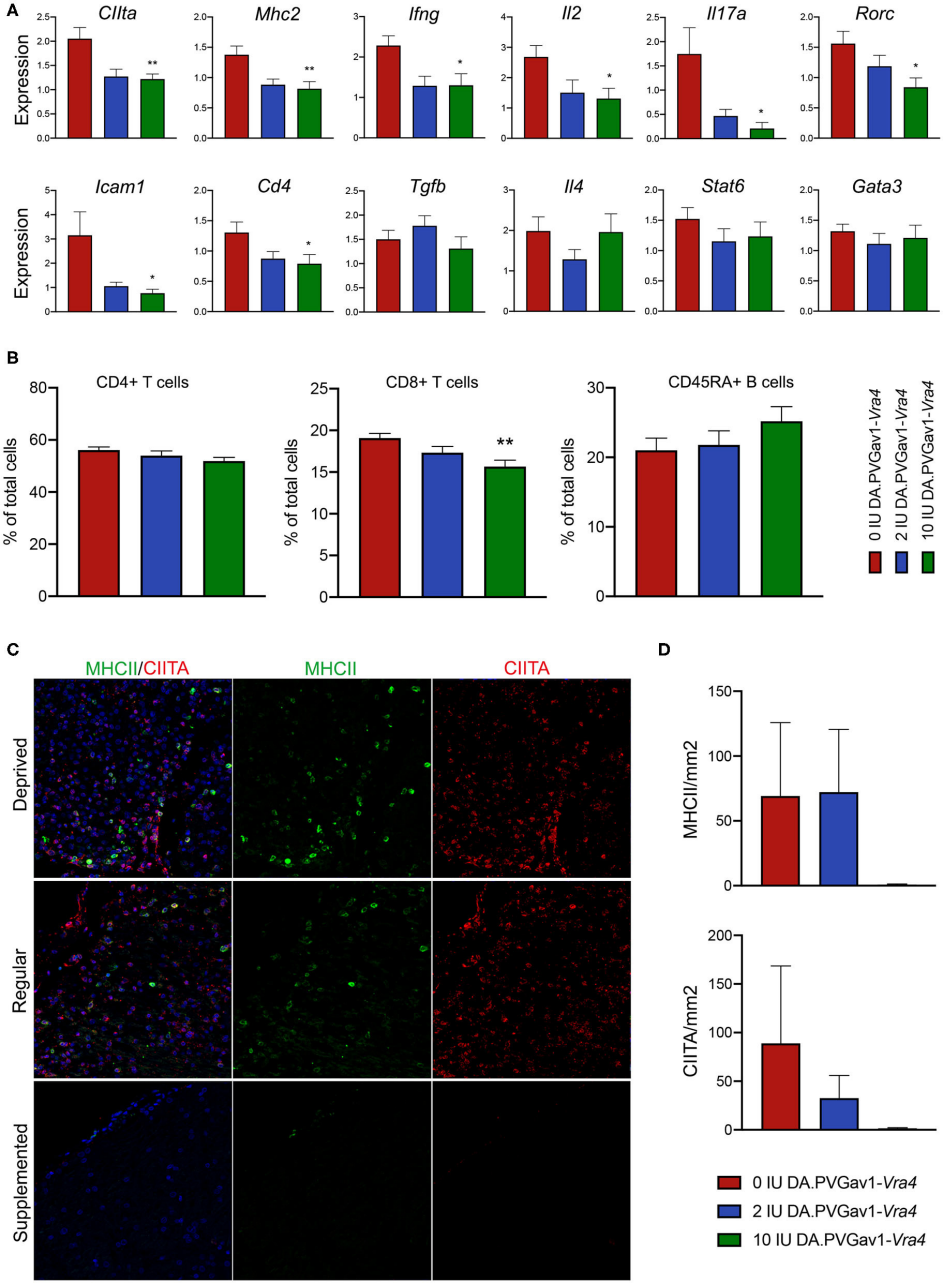
Impact of vitamin D on the peripheral immune response and expression of MHC class II and CIITA in the CNS of DA.PVGav1-*Vra4* congenic rats. **(A)** qPCR performed on mRNA isolated from local draining lymph nodes and **(B)** Flow cytometry performed on d7 LN single cell suspension of the congenic rats subjected to either: (i) vitamin D supplemented, (ii) deprived, or (iii) regular diet (data based on 2 pooled independent experiments with similar outcome). **(A)** Graphs presenting mRNA levels of indicated targets (*n* = 8-12 per diet group). Vitamin D supplementation downregulates Th1 type response in the congenic strain. Relative expression was calculated in relation to the mean of housekeeping gene *Rpl19* using 2-ΔΔCT method. **(B)** Graphs presenting total cell counts of CD4+ and CD8+ T cells as well as CD45RA+ B cells (*n* = 12 per diet group). Amount of CD8+ cells is significantly downregulated upon vitamin D supplementation. **(A,B)** 2 independent experiments with similar outcome pooled. **(C)** Immuno-co-targeting of MHC class II (green) and CIITA (red) in the same CNS tissue samples (*n* = 4-6 per diet group) as in the Figure 3. Vitamin D supplementation tends to downregulate both MHC class II and CIITA in the congenic strain as shown in the quantification in **(D)**. Error bars represent SEM. Statistics were calculated using one-way ANOVA with Tukey correction for multiple testing (*p < 0.05), (**p < 0.001). Statistical significances refer to comparison between vitamin D deprived and supplemented DA.PVGav1-*Vra4*.

DA rats mount a strong peripheral immune response against MOG approximately one week after immunization, which subsequently leads to recruitment of inflammatory cells into CNS and clinical manifestation of the disease (28). To that end, we performed transcriptional analyses on d7 LN obtained from the rats fed with either regular, vitamin D supplemented or vitamin D deprived diet. The analysis revealed vitamin D supplementation-associated significant downregulation of Ifng and Il2, cytokines associated with the pro-inflammatory Th1 immune response, in both congenic and DA rats (Figure 4A, Supplementary Figure 1). We could also observe reduced expression of *Il17a* and *Rorc*, genes important for the Th17 immune response, upon vitamin D supplementation in both the congenic and parental DA strain (Figure 4A, Supplementary Figure 1). Further, adhesion molecule *Icam1* and the T-cell co-receptor *Cd4* were significantly downregulated in DA.PVG^av1^-*Vra4* fed with vitamin D supplemented diet (Figure 4A). In contrast to DA strain, although downregulating Th1 and Th17 genes, vitamin D did not seem to influence the Th2 type immune response in DA.PVG^av1^-*Vra4* rats, as demonstrated by comparable levels of *Tgfb, Il4, Stat6*, and *Gata3* in all three diet groups (Figure 4A, Supplementary Figure 1). Immune-profile of the supplemented group was additionally characterized by significantly reduced number of CD8+ T cells, as revealed by flow cytometry (Figure 4B). Finally, in line with lower expression levels of *CIIta* and *MhcII* in the d7 LN (Figure 4A), we could observe reduced protein expression of MHC class II and CIITA in the CNS of the congenic rats fed with vitamin D supplemented diet (Figures 4C,D).

## Discussion

The HLA class II region is the key susceptibility locus in MS that has the highest influence on the overall disease risk of all identified susceptibility genes (29, 30). *CIITA* encodes a transcription factor, the protein CIITA, a major regulator of HLA class II (31). Notably, genetic variances in CIITA correlate with differential expression of HLA class II, suggested to modulate susceptibility to complex inflammatory diseases (12, 32).

*CIITA* gene expression is regulated by epigenetic mechanisms such as DNA methylation and histone modifications (10). These modifications can be influenced by environmental factors, which is underpinned by the presence of a functional VDRE in the promoter region of the main MS-linked HLA-DRB1^*^15:01 allele (16). Moreover, VDR binding has been found enriched near autoimmune risk genes, which additionally implicates vitamin D in modulation of susceptibility to autoimmune diseases (33). In addition to the functional VDRE in the HLA class promotor, *CIITA* contains putative VDREs suggesting that vitamin D may have a direct effect on CIITA, and not only on HLA class II (34).

Numerous EAE studies attest benefits of vitamin D, nevertheless, data reporting differential response to this environmental factor in the context of genetic variations within the *Vra4* region are still missing. To that end, our previous studies in DA rats (22, 23) were extended toward assessing effects of the same supplementation regimen on EAE in DA.PVG^av1^-*Vra4* strain, in which the *Vra4* locus originates from the PVG^av1^ strain. Notably, we have previously observed a marked attenuation of MOG-induced EAE in inbred juvenile/adolescent DA rats using five times higher than regular amount of vitamin D in the diet, which was reflected in a less severe neuroinflammation and demyelination, accompanied by downregulation of the peripheral immune response. Interestingly, although being protective in both DA and the congenic strain, vitamin D-mediated immunomodulation during clonal T cell expansion targeted different cell populations in the local draining lymph nodes of those two strains. While downregulating genes associated with a pro-inflammatory Th1 response such as *Il2, Icam1* and *Ifng*, vitamin D did not seem to have any effect on the Th2-like response (*Gata3, Stat6, Il4, Tgfb*) in the congenic strain, unlike previously observed inducing of an anti-inflammatory response in DA rats (23). Further, besides lower expression level of *Rorc* (23), suppressive effect of vitamin D on Th17 cells in the congenic rats was additionally supported by downregulation of *Il17a*, which was also observed in DA rats fed with the supplemented diet.

Substantial evidence from patients with MS points to a role of CD8+ T cells in pathogenesis of the demyelinating disease (35). Moreover, a recent study reported that CD8 T cells play an important role even in pathogenesis of EAE by exacerbating neuroinflammation (36, 37). Notably, the supplementation regimen used in our studies significantly reduced amount of CD8+ T cells in the congenic d7 LN in contrast to DA, in which exclusively CD45RA+ B cell counts were affected by vitamin D (23). Downregulation of *Il2* and *Ifng* coinciding with the lower CD8+ T cell count in the vitamin D supplemented congenic group is in accordance with a previous study showing that IL-2 and IFNγ producing CD4+ T-cells are required for the generation of effective cytotoxic T cell lymphocyte (CTL) immunity to infection (38).

Although an additional amelioration of essentially mild and comparable disease course in all 3 diet groups of congenic rats was less probable, diet content of vitamin D-related differences were apparent in the subclinical phenotype. Thus, despite generally reduced MOG-mediated inflammatory infiltration and demyelination in the congenic strain, we could still note clear tendencies toward overall better preservation of the CNS parenchyma in the supplemented group. Nevertheless, an intact myelination in absence of inflammatory infiltration was also observed in single congenic rats subjected either to vitamin D deprived- or regular diet, which conditioned heterogeneity of the two latter groups. In line with reduced amount of CD8+ T cells in the local draining lymph nodes and protective immunomodulation reflected in downregulation of genes essential for the Th1/Th17-like response, we could detect a trend toward a lower T cell infiltration rate in the CNS of DA.PVG^av1^-*Vra4* rats subjected to vitamin D supplemented diet. Moreover, CNS parenchyma of the supplemented group was virtually free from APP+ depositions and ED1+ infiltrating macrophages/activated microglia. The supplementation phenotype was further characterized by less abundant MHC class II+ and CIIta+ cells, underpinned by significant peripheral downregulation of *Mhc class II* and *Mhct2a*, prior to clinical onset of the disease. Lesion-associated MHC II-expressing antigen presenting cells (APCs) (39) are critical players in perpetuation of the inflammatory milieu in MS (40). It is therefore tempting to speculate that supplementation-associated downregulation of pro-inflammatory mediators as well as reduced amount, and possibly even capacity, of APCs to present myelin epitopes to autoreactive T cells, is primarily mediated by the VRA4 allelic region.

Assessing efficacy of vitamin D in amelioration of MS-like neuroinflammation in the context of naturally occurring allelic differences in the *Vra4* locus revealed differential responsiveness to this environmental factor between DA and PVG strain. Considering the impact on MHC class II and CIIta expression on both transcriptional and protein level in the congenic rats fed with the supplemented diet, our data primarily underline relevance of *Mhc2ta* for vitamin D-mediated modulation of EAE. Nevertheless, further studies are required for revealing involvement of other genes in the *Vra4* locus in regulation of vitamin D-mediated disease modulation, such as *Socs1, Dexi*, and *Clec16a* (11). So far it has been shown that vitamin D promotes negative feedback regulation of Toll like receptor (TLR) signaling by inhibiting microRNA-155 mediated downregulation of suppressor of cytokine signaling 1 (SOCS1) in macrophages *in vitro*, which in turn ameliorates inflammation (41). Vitamin D also downregulates CLEC16A and HLA class II expression in human monocyte-derived dendritic cells (42), which is in line with presence of a vitamin D receptor binding site in the respective genes (33).

Taken together, by showing different impact of vitamin D on the CNS pathology and peripheral immune response during EAE in DA.PVG^av1^-*Vra4* congenic rats compared to the parental strains, our data emphasize relevance of naturally occurring allelic differences in the *Vra4* locus/*Mhc2ta* for efficacy of vitamin D in modulation of MS-like neuroinflammation, and potentially even MS.

## Data Availability Statement

The raw data supporting the conclusions of this article will be made available by the authors, without undue reservation.

## Ethics Statement

The animal study was reviewed and approved by Swedish National Board for Laboratory Animals.

## Author Contributions

MZA and MZ conceived and designed the study and wrote the manuscript with input from the other authors. SH, SA, CD, RB, MH, VV, SG, MZ, and MZA performed the experiments. SH, SA, CD, MZ, and MZA analyzed and interpreted the results. TO, FP, and MJ reviewed the study. All authors contributed to the article and approved the submitted version.

## Conflict of Interest

The authors declare that the research was conducted in the absence of any commercial or financial relationships that could be construed as a potential conflict of interest.

## Supplementary Material

Supplementary Figure 1 qPCR analyses performed on mRNA isolated from local draining lymph nodes of DA rats 7 days p.i subjected to either: (i) vitamin D supplemented, (ii) deprived, or (iii) regular diet. Graphs present mRNA levels of indicated targets (*n* = 5–7 rats per diet group). Vitamin D supplementation downregulates Th1 type response and upregulates Th2 type response in the DA strain. Relative expression was calculated in relation to the mean of housekeeping gene Rpl19 using 2-ΔΔCT method. Experimental design including EAE induction and diet regimen as previously described in (22, 23) as well as in the Materials and methods section of the present study.

## References

[1] Filippi M Bar-Or A Piehl F Preziosa P Solari A Vukusic S Multiple sclerosis. Nat Rev Dis Primers. (2018) 4:43 10.1038/s41572-018-0041-4 30410033

[2] Compston A Coles A. Multiple sclerosis. Lancet. (2008) 372:1502–17. 10.1016/S0140-6736(08)61620-7 18970977

[3] International Multiple Sclerosis Genetics C. Multiple sclerosis genomic map implicates peripheral immune cells and microglia in susceptibility. Science. (2019) 365(6460). 10.1126/science.aav7188 31604244 PMC7241648

[4] Nerup J Platz P Andersen OO Christy M Lyngsoe J Poulsen JE HL-A antigens and diabetes mellitus. Lancet. (1974) 2:864–6. 10.1016/S0140-6736(74)91201-X 4137711

[5] Hirschhorn JN Genetic epidemiology of type 1 diabetes. Pediatr Diabetes. (2003) 4:87–100. 10.1034/j.1399-5448.2001.00013.x 14655265

[6] Koizumi K Okamoto H Iikuni N Nakamura T Kawamoto M Momohara S. Single nucleotide polymorphisms in the gene encoding the major histocompatibility complex class II transactivator (CIITA) in systemic lupus erythematosus. Ann Rheum Dis. (2005) 64:947–50. 10.1136/ard.2004.025767 15897313 PMC1755521

[7] Gregersen PK Silver J Winchester RJ. The shared epitope hypothesis. An approach to understanding the molecular genetics of susceptibility to rheumatoid arthritis. Arthritis Rheum. (1987) 30:1205–13. 10.1002/art.1780301102 2446635

[8] Huizinga TW Amos CI van der Helm-van Mil AH Chen W van Gaalen FA Jawaheer D. Refining the complex rheumatoid arthritis phenotype based on specificity of the HLA-DRB1 shared epitope for antibodies to citrullinated proteins. Arthritis Rheum. (2005) 52:3433–8. 10.1002/art.21385 16255021

[9] Mignot E Lin L Rogers W Honda Y Qiu X Lin X. Complex HLA-DR and -DQ interactions confer risk of narcolepsy-cataplexy in three ethnic groups. Am J Hum Genet. (2001) 68:686–99. 10.1086/318799 11179016 PMC1274481

[10] Wright KL Ting JP. Epigenetic regulation of MHC-II and CIITA genes. Trends Immunol. (2006) 27:405–12. 10.1016/j.it.2006.07.007 16870508

[11] Berge T Leikfoss IS Harbo HF. From identification to characterization of the multiple sclerosis susceptibility gene CLEC16A. Int J Mol Sci. (2013) 14:4476–97. 10.3390/ijms14034476 23439554 PMC3634488

[12] Swanberg M Lidman O Padyukov L Eriksson P Akesson E Jagodic M. MHC2TA is associated with differential MHC molecule expression and susceptibility to rheumatoid arthritis, multiple sclerosis and myocardial infarction. Nat Genet. (2005) 37:486–94. 10.1038/ng1544 15821736

[13] Weissert R Wallstrom E Storch MK Stefferl A Lorentzen J Lassmann H. MHC haplotype-dependent regulation of MOG-induced EAE in rats. J Clin Invest. (1998) 102:1265–73. 10.1172/JCI3022 9739061 PMC509110

[14] Lundberg C Lidman O Holmdahl R Olsson T Piehl F. Neurodegeneration and glial activation patterns after mechanical nerve injury are differentially regulated by non-MHC genes in congenic inbred rat strains. J Comp Neurol. (2001) 431:75–87. 10.1002/1096-9861(20010226)431:1<75::AID-CNE1056>3.0.CO;2-M 11169991

[15] Harnesk K Swanberg M Ockinger J Diez M Lidman O Wallstrom E. Vra4 congenic rats with allelic differences in the class II transactivator gene display altered susceptibility to experimental autoimmune encephalomyelitis. J Immunol. (2008) 180:3289–96. 10.4049/jimmunol.180.5.3289 18292553

[16] Ramagopalan SV Maugeri NJ Handunnetthi L Lincoln MR Orton SM Dyment DA. Expression of the multiple sclerosis-associated MHC class II Allele HLA-DRB1^*^1501 is regulated by vitamin D. PLoS Genet. (2009) 5:e1000369. 10.1371/journal.pgen.1000369 19197344 PMC2627899

[17] Waubant E Lucas R Mowry E Graves J Olsson T Alfredsson L. Environmental and genetic risk factors for MS: an integrated review. Ann Clin Transl Neurol. (2019) 6:1905–22. 10.1002/acn3.50862 31392849 PMC6764632

[18] Ascherio A Munger KL Simon KC. Vitamin D and multiple sclerosis. Lancet Neurol. (2010) 9:599–612. 10.1016/S1474-4422(10)70086-7 20494325

[19] Souberbielle JC Body JJ Lappe JM Plebani M Shoenfeld Y Wang TJ. Vitamin D and musculoskeletal health, cardiovascular disease, autoimmunity and cancer: Recommendations for clinical practice. Autoimmun Rev. (2010) 9:709–15. 10.1016/j.autrev.2010.06.009 20601202

[20] Cocco E Meloni A Murru MR Corongiu D Tranquilli S Fadda E. Vitamin D responsive elements within the HLA-DRB1 promoter region in Sardinian multiple sclerosis associated alleles. PLoS ONE. (2012) 7:e41678. 10.1371/journal.pone.0041678 22848563 PMC3404969

[21] Zierold C Darwish HM DeLuca HF. Two vitamin D response elements function in the rat 1,25-dihydroxyvitamin D 24-hydroxylase promoter. J Biol Chem. (1995) 270:1675–8. 10.1074/jbc.270.4.1675 7829502

[22] Zeitelhofer M Adzemovic MZ Gomez-Cabrero D Bergman P Hochmeister S N'Diaye M. Functional genomics analysis of vitamin D effects on CD4+ T cells *in vivo* in experimental autoimmune encephalomyelitis. Proc Natl Acad Sci USA. (2017) 114:E1678–E87. 10.1073/pnas.1615783114 28196884 PMC5338504

[23] Adzemovic MZ Zeitelhofer M Hochmeister S Gustafsson SA Jagodic M. Efficacy of vitamin D in treating multiple sclerosis-like neuroinflammation depends on developmental stage. Exp Neurol. (2013) 249:39–48. 10.1016/j.expneurol.2013.08.002 23954214

[24] Amor S Groome N Linington C Morris MM Dornmair K Gardinier MV. Identification of epitopes of myelin oligodendrocyte glycoprotein for the induction of experimental allergic encephalomyelitis in SJL and Biozzi AB/H mice. J Immunol. (1994) 153:4349–56. 7525700

[25] Storch MK Stefferl A Brehm U Weissert R Wallstrom E Kerschensteiner M. Autoimmunity to myelin oligodendrocyte glycoprotein in rats mimics the spectrum of multiple sclerosis pathology. Brain Pathol. (1998) 8:681–94. 10.1111/j.1750-3639.1998.tb00194.x 9804377 PMC8098227

[26] Adzemovic MZ Ockinger J Zeitelhofer M Hochmeister S Beyeen AD Paulson A. Expression of Ccl11 associates with immune response modulation and protection against neuroinflammation in rats. PLoS ONE. (2012) 7:e39794. 10.1371/journal.pone.0039794 22815714 PMC3397980

[27] Gold R Hartung HP Toyka KV. Animal models for autoimmune demyelinating disorders of the nervous system. Mol Med Today. (2000) 6:88–91. 10.1016/S1357-4310(99)01639-1 10652482

[28] Thessen Hedreul M Gillett A Olsson T Jagodic M Harris RA. Characterization of Multiple Sclerosis candidate gene expression kinetics in rat experimental autoimmune encephalomyelitis. J Neuroimmunol. (2009) 210:30–9. 10.1016/j.jneuroim.2009.02.010 19269041

[29] Ramagopalan SV Ebers GC. Genes for multiple sclerosis. Lancet. (2008) 371:283–5. 10.1016/S0140-6736(08)60145-2 18294983

[30] Herrera BM Cader MZ Dyment DA Bell JT Ramagopalan SV Lincoln MR. Follow-up investigation of 12 proposed linkage regions in multiple sclerosis. Genes Immun. (2006) 7:366–71. 10.1038/sj.gene.6364308 16738670

[31] Handunnetthi L Ramagopalan SV Ebers GC Knight JC. Regulation of major histocompatibility complex class II gene expression, genetic variation and disease. Genes Immun. (2010) 11:99–112. 10.1038/gene.2009.83 19890353 PMC2987717

[32] Jimenez-Ferrer I Jewett M Tontanahal A Romero-Ramos M Swanberg M. Allelic difference in Mhc2ta confers altered microglial activation and susceptibility to alpha-synuclein-induced dopaminergic neurodegeneration. Neurobiol Dis. (2017) 106:279–90. 10.1016/j.nbd.2017.07.016 28736195

[33] Ramagopalan SV Heger A Berlanga AJ Maugeri NJ Lincoln MR Burrell A. A ChIP-seq defined genome-wide map of vitamin D receptor binding: associations with disease and evolution. Genome Res. (2010) 20:1352–60. 10.1101/gr.107920.110 20736230 PMC2945184

[34] Bronson PG Caillier S Ramsay PP McCauley JL Zuvich RL De Jager PL. CIITA variation in the presence of HLA-DRB1^*^1501 increases risk for multiple sclerosis. Hum Mol Genet. (2010) 19:2331–40. 10.1093/hmg/ddq101 20211854 PMC2865376

[35] Babbe H Roers A Waisman A Lassmann H Goebels N Hohlfeld R. Clonal expansions of CD8(+) T cells dominate the T cell infiltrate in active multiple sclerosis lesions as shown by micromanipulation and single cell polymerase chain reaction. J Exp Med. (2000) 192:393–404. 10.1084/jem.192.3.393 10934227 PMC2193223

[36] Willis CM Nicaise AM Menoret A Ryu JK Mendiola AS Jellison ER. Extracellular vesicle fibrinogen induces encephalitogenic CD8+ T cells in a mouse model of multiple sclerosis. Proc Natl Acad Sci USA. (2019) 116:10488–93. 10.1073/pnas.1816911116 31068461 PMC6535008

[37] Wagner CA Roque PJ Mileur TR Liggitt D Goverman JM. Myelin-specific CD8+ T cells exacerbate brain inflammation in CNS autoimmunity. J Clin Invest. (2020) 130:203–13. 10.1172/JCI132531 31573979 PMC6934187

[38] Yang Y Xiang Z Ertl HC Wilson JM. Upregulation of class I major histocompatibility complex antigens by interferon gamma is necessary for T-cell-mediated elimination of recombinant adenovirus-infected hepatocytes *in vivo*. Proc Natl Acad Sci USA. (1995) 92:7257–61. 10.1073/pnas.92.16.7257 7638177 PMC41318

[39] Bo L Mork S Kong PA Nyland H Pardo CA Trapp BD. Detection of MHC class II-antigens on macrophages and microglia, but not on astrocytes and endothelia in active multiple sclerosis lesions. J Neuroimmunol. (1994) 51:135–46. 10.1016/0165-5728(94)90075-2 8182113

[40] Chastain EM Duncan DS Rodgers JM Miller SD. The role of antigen presenting cells in multiple sclerosis. Biochim Biophys Acta. (2011) 1812:265–74. 10.1016/j.bbadis.2010.07.008 20637861 PMC2970677

[41] Chen Y Liu W Sun T Huang Y Wang Y Deb DK. 1,25-Dihydroxyvitamin D promotes negative feedback regulation of TLR signaling via targeting microRNA-155-SOCS1 in macrophages. J Immunol. (2013) 190:3687–95. 10.4049/jimmunol.1203273 23436936 PMC3608760

[42] van Luijn MM Kreft KL Jongsma ML Mes SW Wierenga-Wolf AF van Meurs M. Multiple sclerosis-associated CLEC16A controls HLA class II expression via late endosome biogenesis. Brain. (2015) 138(Pt 6):1531–47. 10.1093/brain/awv080 25823473 PMC4614123

