# Supplementary figures and images for: Effect of Vitamin D on Experimental Autoimmune Neuroinflammation Is Dependent on Haplotypes Comprising Naturally Occurring Allelic Variants of CIITA (Mhc2ta)
Source: Front Neurol. 2020 Nov 13;11:600401. doi: 10.3389/fneur.2020.600401 (PMC7693436; doi:10.3389/fneur.2020.600401)

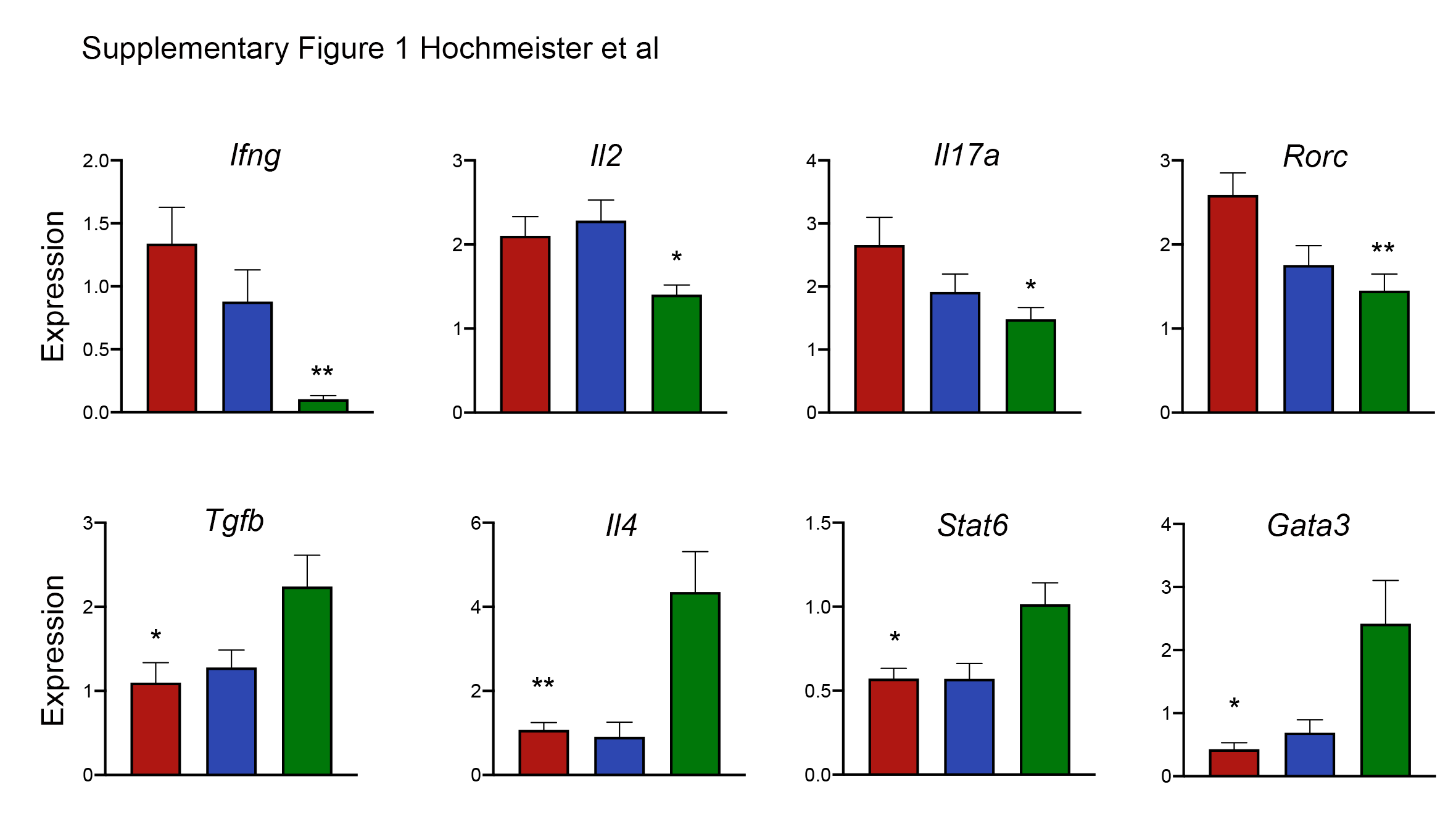

Supplement: Supplementary Figure 1 — qPCR analyses performed on mRNA isolated from local draining lymph nodes of DA rats 7 days p.i subjected to either: (i) vitamin D supplemented, (ii) deprived, or (iii) regular diet. Graphs present mRNA levels of indicated targets (n = 5–7 rats per diet group). Vitamin D supplementation downregulates Th1 type response and upregulates Th2 type response in the DA strain. Relative expression was calculated in relation to the mean of housekeeping gene Rpl19 using 2-ΔΔCT method. Experimental design including EAE induction and diet regimen as previously described in (22, 23) as well as in the Materials and methods section of the present study. [file Image_1.TIF]
